# Cognitive Function and Brain Atrophy Predict Non-pharmacological Efficacy in Dementia: The Mihama-Kiho Scan Project2

**DOI:** 10.3389/fnagi.2018.00087

**Published:** 2018-04-12

**Authors:** Ken-ichi Tabei, Masayuki Satoh, Jun-ichi Ogawa, Tomoko Tokita, Noriko Nakaguchi, Koji Nakao, Hirotaka Kida, Hidekazu Tomimoto

**Affiliations:** ^1^Department of Dementia Prevention and Therapeutics, Graduate School of Medicine, Mie University, Tsu, Japan; ^2^Department of Neurology, Graduate School of Medicine, Mie University, Tsu, Japan; ^3^Yamaha Music Foundation, Tokyo, Japan; ^4^Department of Health and Welfare, Mihama Town Hall, Mihama, Japan; ^5^Department of Health and Welfare, Kiho Town Hall, Kiho, Japan; ^6^Department of Neurosurgery, Kinan Hospital, Mihama, Japan

**Keywords:** dementia, exercise with music, cognitive dysfunction, voxel-based morphometry, atrophy, frontal lobe

## Abstract

We aimed to determine whether neuropsychological deficits and brain atrophy could predict the efficacy of non-pharmacological interventions. Forty-six participants with mild-to-moderate dementia were monitored for 6 months; 25 underwent an intervention involving physical exercise with music, and 21 performed cognitive stimulation tasks. Participants were categorized into improvement (IMP) and no-IMP subgroups. In the exercise-with-music group, the no-IMP subgroup performed worse than the IMP subgroup on the Rivermead Behavioural Memory Test at baseline. In the cognitive-stimulation group, the no-IMP subgroup performed worse than the IMP subgroup on Raven’s Colored Progressive Matrices and the cognitive functional independence measure at baseline. In the no-IMP subgroup, voxel-based morphometric analysis at baseline revealed more extensive gray matter loss in the anterior cingulate gyrus and left middle frontal gyrus in the exercise-with-music and cognitive-stimulation groups, respectively. Participants with mild-to-moderate dementia with cognitive decline and extensive cortical atrophy are less likely to show improved cognitive function after non-pharmaceutical therapy.

## Introduction

The World Alzheimer Report has estimated that there were 46.8 million people with dementia worldwide in 2015, and that this number will reach 131.5 million by 2050 (Prince et al., 2015). However, pharmacological treatments are relatively ineffective in halting the progression of dementia. The lack of pharmacological options (e.g., vaccines or disease-modifying agents) has shifted the focus of researchers toward non-pharmacological treatments for dementia, such as cognitive training, aerobic physical exercise, and music therapy (Gates and Sachdev, 2014; Groot et al., 2016; Zhang et al., 2017).

Accumulating evidence suggests that non-pharmacological treatment may maintain or decrease the rate of cognitive decline in adults with mild cognitive impairment and early stage dementia (Rodakowski et al., 2015). Additionally, the protective effects of aerobic physical exercise against the development and/or progression of dementia have been well documented (Laurin et al., 2001; Abbott et al., 2004; Ravaglia et al., 2008; Erickson et al., 2011). Additional studies have suggested that physical exercise combined with cognitive training exerts a greater positive impact on cognitive function than physical exercise alone (Fabre et al., 2002; Oswald et al., 2006; Shatil, 2013; Satoh et al., 2014, 2017; Tabei et al., 2017). In older adults, physical ExM induces greater positive effects on visuospatial function and leads to more extensive neuroanatomical changes (Tabei et al., 2017) than exercise alone. In participants with mild-to-moderate dementia, ExM also exerts greater positive effects on cognitive function and activities of daily living than CS using portable game consoles or drills (e.g., easy calculations, mazes, and mistake-searching in pictures) (Satoh et al., 2017).

Despite recent advancements, few studies have focused on predicting outcomes following non-pharmacological treatment. Knowledge of such predictors may allow clinicians to modify factors that are likely to attenuate the effects of the intervention and to provide more targeted treatment (Hsu et al., 2017). Indeed, previous studies have demonstrated that clinical and demographic factors can influence the effect of non-pharmacological activities on cognition and on the behavioral and psychological symptoms of dementia (Särkämö et al., 2016; Hsu et al., 2017). However, the neuropsychological factors influencing non-pharmacological treatment, as well as the neural basis of its efficacy, remain unknown. Furthermore, no randomized controlled trials have compared such effects among different non-pharmacological treatment options.

Therefore, the purpose of the present study was to determine whether neuropsychological factors and regional brain atrophy [as measured via voxel-based morphometry (VBM)] can predict the rate of improvement following non-pharmacological treatment in participants with mild-to-moderate dementia and to compare the effects of different non-pharmacological interventions.

## Materials and Methods

### Participants

The present study included participants with mild-to-moderate dementia who had utilized nursing services, such as day care or group homes, in the towns of Mihama and Kiho, which are situated at the southern end of the Kii peninsula in Japan. This study received approval from the Kinan Hospital Research Ethics Committee, and conformed to the tenets of the Declaration of Helsinki, and all participants provided written informed consent. This study was registered at UMIN-CTR (UMIN000017066) on April 7, 2015. The present study included the same participants as a previous study, which investigated the effect of non-pharmacological interventions in participants with mild-to-moderate dementia (Satoh et al., 2017). The inclusion criteria were as follows: (a) current diagnosis of intractable dementia by neurological specialists, based on ICD-10 diagnostic criteria (World Health Organization, 1993); (b) score between 16 and 26 on the MMSE; (c) stable physical and psychological condition; and (d) preserved hearing, visual acuity, and physical movement sufficient to enable participation in the intervention. Participants were excluded if they met any of the following criteria: (a) presence of chronic debilitating disease, such as malignancy or infection; (b) presence of severe cardiac, respiratory, and/or orthopedic disabilities that would prevent participation in the intervention; (c) presence of paresis or coordination disturbances that would prevent participation in the intervention; and (d) diagnosis of treatable dementia.

Diagnoses of AD were performed in accordance with criteria established by the National Institute of Neurologic Disorders and Stroke/Alzheimer Disease and Related Disorders Association (McKhann et al., 1984). Diagnoses of VaD were performed in accordance with criteria established by the National Institute of Neurological Disorders and Stroke/Association Internationale pour la Recherche et l’Enseignement en Neurosciences (Román et al., 1993).

All participants received treatment with anti-dementia drugs, the most common of which was donepezil hydrochloride. Pharmacological and non-pharmacological activities performed in the nursing-care facilities remained unchanged during the 6-months intervention period. Neuropsychological assessments and brain MRI were performed at baseline and following the intervention.

### Procedures

Detailed descriptions of the procedures for each non-pharmacological intervention and neuropsychological assessment can be found in our previous report (Satoh et al., 2017).

#### Physical Exercise With Music (ExM)

Participants participated in 40-min physical exercise sessions once per week over the 6-months intervention period (total number of sessions: 24). The exercise program was developed by the Yamaha Music Foundation based on a program used in our previous study, which investigated the efficacy of physical exercise combined with music in older adults with normal cognitive function (Satoh et al., 2014; Tabei et al., 2017). The program consisted of muscle training for the upper and lower extremities, hand clapping to music, breath and voice training, and singing. The exercise trainers were professional musicians who also held private licenses as physical trainers with the Yamaha Music Foundation.

#### Cognitive Stimulation (CS)

Participants trained using a program called “Yawaraka Atama Juku (flexible thinking club)” developed by Nintendo Co., Ltd., using a portable game console (Nintendo DS LL, Kyoto, Japan) and performed drills consisting of easy calculations (Kawashima, 2003), mazes, and mistake-searching in pictures. Each session was 40 min long and were conducted once per week over the 6-months intervention period (total sessions: 24). Each session was moderated by nurses, certified care workers, or psychiatric social workers employed at the respective nursing facility.

#### Neuropsychological Assessment

The MMSE (Folstein et al., 1975) and RCPM (Raven, 1947) were used to quantify cognitive function. In addition to an overall score, the RCPM task measures performance time, which reflects the psychomotor speed of the participants. Memory was evaluated using the LM-I/-II subtests of the RBMT (Wilson et al., 1985), which requires immediate and delayed recall of a short story. The RBMT contains four stories of varying difficulty and different word counts. We used different stories for the pre- and post-test periods to avoid the influence of familiarity with story content.

Visuospatial ability was assessed using methods described by Strub and Black (2001). Participants were shown images of cubes and Necker cubes, and were then asked to draw a picture of each. Each drawing was scored by assigning one of four possible grades (0: poor, 1: fair, 2: good, and 3: excellent).

Frontal lobe function was assessed using two tasks: WF and Trail Making Test -A/-B (Partington and Leiter, 1949). The WF test consists of category and letter domains. In the categorical WF task, participants were asked to name as many animals as possible in 1 min. In the letter WF task, participants were asked to name as many objects as possible in 1 min beginning with each of the following four phonemes: ka, sa, ta, and te (Dohi et al., 1992). The average scores for these four phonemes were used for statistical analyses.

The Functional Independence Measure was used to evaluate functional performance regarding activities of daily living. The Functional Independence Measure consists of both motor and cognitive function domains. Motor function is assessed on thirteen items, including eating, dressing, evacuation, urination, and walking. Cognitive function is assessed on five items associated with understanding, expression, and memory. The maximum motor function, cognitive function, and overall Functional Independence Measure scores are 91, 35, and 126, respectively, with higher scores indicating better function. These neuropsychological assessments were administered to participants in both groups before and after the 6-month intervention period.

#### Improvement and No-Improvement Groups

Participants were dichotomized into an IMP or no-IMP subgroup based on MMSE scores following the intervention. Participants with an increased MMSE score of 2 points or more were included in the IMP subgroup, while the remaining participants were included in the no-IMP subgroup. The cut-off of 2 points was determined based on the findings of previous studies, which had shown that changes in MMSE scores of 2 points are beyond the threshold of chance (Morris et al., 1993; Bowie et al., 1999; Ballard et al., 2003).

#### MRI Acquisition

T1-weighted gradient echo MR images were obtained using a 1.5-T MR scanner (Echelon Oval, Hitachi Medical Corporation, Tokyo, Japan). Scan parameters were as follows: repetition time = Shortest (Automatic); echo time = 11 ms; flip angle = 90°; field of view = 230 mm × 230 mm; slice thickness = 4 mm; gapless; in-plane resolution = 0.45 mm × 0.45 mm. Scans were obtained both before and after the 6-month intervention period.

#### MRI Analysis

MRI data were analyzed using SPM12 (Wellcome Trust Centre for Neuroimaging, University College London, London, United Kingdom) implemented in MATLAB R2012a (MathWorks, Natick, MA, United States). In the pre-processing phase, images were set to match the anterior to posterior commissure line using an automated MATLAB script. The images were then visually inspected to check for possible scan issues such as field distortion and movement artifacts. Reoriented images were corrected for intensity inhomogeneity and segmented into GM, white matter, cerebrospinal fluid, and other tissues outside the brain using SPM12 tissue probability maps. The images were registered to the East Asian Brains International Consortium for Brain Mapping space template via affine regularization. We created a population-specific template using the SPM12 DARTEL template procedure to directly compare data between the IMP and no-IMP subgroups. We investigated group differences in GM volume, as well as the relationship between neuropsychological assessment results and GM at the whole-brain level. High-dimensional DARTEL was used to create non-linear, modulated-normalized GM and white matter images, which were smoothed using a Gaussian kernel of 8 mm FWHM (full-width at half-maximum). For whole-brain and multiple regression analyses, we assessed the statistical significance at a voxel threshold of *p* < 0.005 (uncorrected), within contiguous clusters of at least 20 voxels. We obtained both MNI and Talairach coordinates to detect the anatomical regions of the clusters. We used a transform from Matthew Brett^1^ to convert MNI coordinates to Talairach coordinates, and Talairach Client 2.4.3 (Lancaster et al., 2000) was used to identify the anatomical regions corresponding to Talairach coordinates.

#### Statistical Analyses

Differences in demographic variables and neuropsychological assessment results between the IMP and no-IMP subgroups were analyzed using independent *t*-tests for continuous data, chi-square tests for dichotomous data, and Mann–Whitney *U* tests for non-parametric data. Differences of *p* < 0.05 were considered statistically significant. Statistical analyses were performed using IBM SPSS Statistics software version 20 (IBM Corp., Armonk, NY, United States).

## Results

### Participant Characteristics

Participants who participated in more than 75% of all sessions (more than 18) and completed the neuropsychological and MRI assessments before and after the intervention were included in the final analysis. Following exclusion of the remaining participants, the ExM and CS groups included 25 and 21 participants, respectively (ExM: AD = 21; VaD = 4; CS: AD = 18, VaD = 3) (**Table 1**). Prior to the intervention, there were no significant differences in sex ratio, dementia type, age, years of education, or MMSE scores between the ExM and CS groups. However, among participants in both ExM and CS groups, MMSE scores were significantly worse in the no-IMP subgroup than in the IMP subgroup at the 6-month follow-up (*p* < 0.001) (**Table 1**).

**Table 1:** Characteristics of study participants.

Characteristics	Total IMP	No-IMP	*p*-value	ExM IMP	No-IMP	*p*-value	CS IMP	No-IMP	*p*-value
No. of participants	18	28	0.183	11	14	0.690	7	14	0.189
Sex (F/M)	17/1	27/1	0.174	11/0	14/0	0.690	6/1	13/1	0.167
Dementia type (AD/VaD)	17/1	22/6	0.522	10/1	11/3	1.000	7/0	11/3	0.481
Age, y, mean (*SD*)	89.4 (4.9)	86.4 (5.1)	0.053	89.1 (5.7)	85.6 (5.8)	0.143	89.9 (3.6)	87.1 (4.4)	0.178
Years of education, mean (*SD*)	7.4 (1.9)	8.0 (2.0)	0.135	7.3 (1.6)	7.1 (2.3)	0.609	7.6 (2.4)	8.9 (1.3)	0.287
MMSE, mean (*SD*)	20.5 (2.9)	20.8 (4.0)	0.726	20.1 (2.5)	20.6 (3.7)	0.716	21.1 (3.6)	21.0 (4.3)	0.913
Change in MMSE score at the 6-month follow up, mean (*SD*)	3.8 (1.6)	-1.5 (2.1)	<0.001	4.4 (1.6)	-1.5 (2.1)	<0.001	3.0 (1.4)	-1.4 (2.1)	<0.001

*IMP, improvement; no-IMP, no improvement; ExM, exercise plus music intervention; CS, cognitive stimulation intervention; AD, Alzheimer’s disease; VaD, vascular dementia; MMSE, Mini Mental State Examination.*

### Neuropsychological Assessments

Baseline cube and Necker cube scores were significantly worse in the ExM group than in the CS group (*p* = 0.022, 0.01), although no significant differences were observed in the other neuropsychological assessments.

Participants in the no-IMP subgroup had significantly poorer baseline scores on the RCPM, LM-I section of the RBMT, and cognitive functional independence than those in the IMP subgroup (*p* = 0.04, 0.004, 0.033) (**Table 2**). Furthermore, baseline scores on the LM-I subtest were significantly worse in the no-IMP subgroup than the IMP subgroup (*p* = 0.025) of the ExM participants. In the CS group, baseline scores on the RCPM test and cognitive functional independence measure were significantly worse in the no-IMP subgroup than in the IMP subgroup (*p* = 0.034, 0.025, respectively) (**Table 2**).

**Table 2:** Baseline neuropsychological performance in the IMP and no-IMP subgroups.

	Total IMP	No-IMP	*p*-value	ExM IMP	No-IMP	*p*-value	CS IMP	No-IMP	*p*-value
MMSE	20.5 (2.9)	20.8 (4.0)	0.726	20.1 (2.5)	20.6 (3.7)	0.716	21.1 (3.6)	21.0 (4.3)	0.913
RCPM (score)	20.7 (5.3)	16.8 (6.4)	0.040	20.2 (6.0)	17.7 (7.3)	0.373	21.4 (4.3)	15.9 (5.6)	0.034
RCPM (time)	755.3 (497.1)	780.7 (782.0)	0.497	747.6 (471.6)	1054.3 (1041.2)	0.709	766.3 (570.1)	507.1 (157.2)	0.172
LM-I	5.4 (2.6)	3.1 (2.6)	0.004	5.2 (1.7)	3.2 (2.5)	0.025	5.9 (3.7)	3.0 (2.7)	0.059
LM-II	2.8 (3.2)	2.0 (2.8)	0.473	2.1 (2.6)	1.9 (2.9)	0.727	3.9 (4.0)	2.1 (2.8)	0.443
Cube	0.7 (0.5)	0.8 (0.5)	0.620	0.6 (0.5)	0.5 (0.5)	0.572	0.7 (0.5)	1.0 (0.4)	0.360
Necker cube	0.3 (0.5)	0.4 (0.5)	0.686	0.3 (0.5)	0.1 (0.4)	0.609	0.4 (0.5)	0.6 (0.5)	0.443
WF (animal)	9.9 (2.2)	8.6 (2.8)	0.099	9.7 (2.2)	8.4 (2.9)	0.209	10.1 (2.4)	8.8 (2.8)	0.172
WF (letter)	5.3 (2.0)	4.8 (2.1)	0.381	5.4 (1.6)	5.0 (1.9)	0.609	5.3 (2.6)	4.6 (2.3)	0.527
TMT-A	350.9 (184.5)	373.5 (182.9)	0.685	381.0 (211.9)	398.2 (158.3)	0.652	303.7 (131.9)	354.1 (203.8)	0.856
FIM (total)	118.6 (8.5)	112.2 (14.4)	0.038	116.9 (10.2)	114.6 (10.7)	0.373	121.3 (4.2)	109.9 (17.4)	0.034
FIM (motor)	85.9 (7.5)	82.4 (11.9)	0.070	85.0 (9.2)	85.5 (10.0)	0.403	87.4 (3.7)	79.2 (13.2)	0.110
FIM (cognition)	32.7 (3.0)	30.6 (4.5)	0.033	31.9 (3.3)	30.5 (4.7)	0.291	33.9 (1.9)	30.6 (4.5)	0.025

*IMP, improvement; no-IMP, no improvement; ExM, exercise plus music intervention; CS, cognitive stimulation intervention; MMSE, Mini Mental State Examination; RCPM, Raven’s Colored Progressive Matrices; LM, Logical Memory; WF, word fluency; TMT, Trail-Making Test; FIM, Functional Independence Measure*.

### MRI Assessments

**FIGURE 1:**
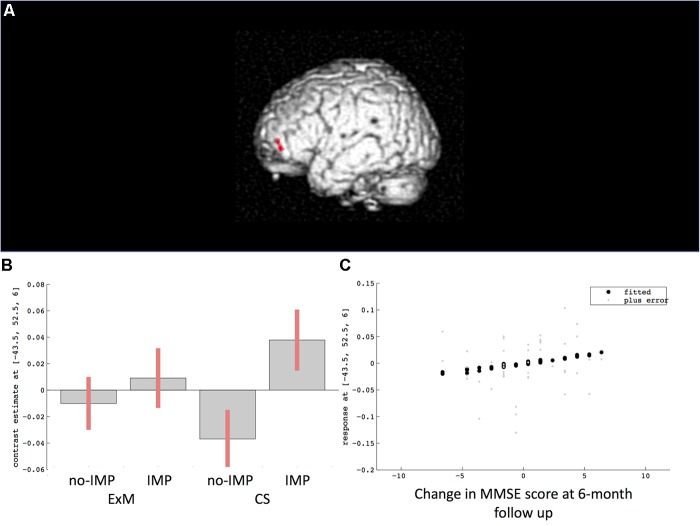
Results of subtraction analysis between the IMP and non-IMP subgroups of the whole cohort **(A,B)**. Correlation between changes in MMSE scores and gray matter (GM) volume **(C)**. IMP, improvement; no-IMP, no-improvement; MMSE, Mini Mental State Examination.

**FIGURE 2:**
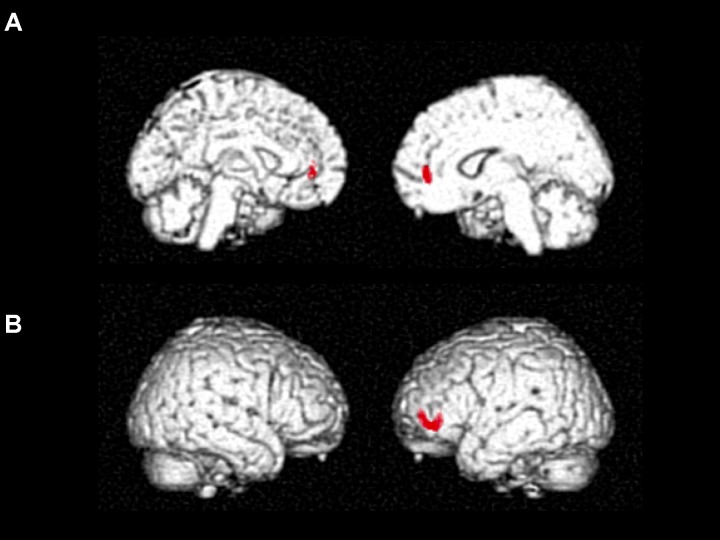
Results of subtraction analysis between the IMP and non-IMP subgroups of the ExM **(A)** and CS groups **(B)**. IMP, improvement; no-IMP, no-improvement; ExM, exercise plus music intervention; CS, cognitive stimulation intervention.

Cube and Necker cube scores were used as covariates. Subtraction analysis revealed more extensive loss of GM in the left middle frontal gyrus in the no-IMP subgroup at baseline, relative to the IMP subgroup (**Figures 1A,B** and **Table 3**). Analysis of MMSE scores at the 6-month follow up revealed that changes in MMSE scores were positively correlated with volume in the left middle frontal gyrus (**Figure 1C** and **Table 3**). Subtraction analysis revealed more extensive GM loss in the anterior cingulate gyrus and left middle frontal gyrus in no-IMP participants in both the ExM (**Figure 2A** and **Table 3**) and CS (**Figure 2B** and **Table 3**) groups at baseline, respectively.

**Table 3:** Cluster sizes, peak locations, and statistical values for regions showing significant pre-intervention differences between the IMP and no-IMP subgroups.

				Talairach coordinates (mm)				
Contrast	L/R	Area	BA	*X*	*Y*	*Z*	*Z*-value	Cluster size in voxels
IMP > no-IMP (Total)	L	Middle Frontal Gyrus	10	-44	46	-4	2.72	49
IMP > no-IMP (ExM)	R	Anterior Cingulate	32	4	44	-2	2.96	209
IMP > no-IMP (CS)	L	Middle Frontal Gyrus	10	-44	50	3	3.20	433

*IMP, improvement; no-IMP, no-improvement; ExM, exercise plus music intervention; CS, cognitive stimulation intervention.*

## Discussion

The present study aimed to determine whether neuropsychological factors and regional brain atrophy can predict the rate of IMP in participants with mild-to-moderate dementia following non-pharmacological intervention. Among ExM participants, the no-IMP subgroup exhibited poorer baseline performance on the LM-I subtest of the RBMT than participants in the IMP subgroup. Among CS participants, the no-IMP subgroup exhibited poorer baseline RCPM and cognitive functional independence measure scores than participants in the IMP subgroup. Such differences may be associated with differences in cognitive resources required to execute each intervention. Proper execution of the ExM protocol requires the participant to remember the exercise patterns from week to week, while proper execution of the CS protocol requires spatial and mathematical abilities that allow understanding of geometric designs and missing pieces, as well as expressive abilities and memory function to complete calculations, mazes, and search for mistakes in images. Although previous studies have indicated that exercise and ExM tasks enhance memory in participants with dementia (Radak et al., 2010; Intlekofer and Cotman, 2013; Satoh et al., 2017), our results suggest that execution of the intervention requires a certain amount of memory function reserve. These findings highlight the importance of early intervention, when cognitive function may be somewhat preserved (Dubois et al., 2016).

Voxel-based morphometry analysis revealed more extensive loss of GM in the anterior cingulate gyrus in no-IMP participants of the ExM group, and in the left middle frontal gyrus in the CS group at baseline relative to that observed in the IMP subgroup. These findings may also have been associated with the execution of each intervention. Previous studies have demonstrated that the anterior cingulate gyrus plays a key role in music processing in participants with dementia (Omar et al., 2011; Fletcher et al., 2015; Jacobsen et al., 2015), while the left middle frontal gyrus is associated with calculation (Maurer et al., 2016) and visual search (Weidner et al., 2009) in CS tasks. Therefore, our results indicate that the extent and location of brain atrophy at baseline may represent the neural basis of task execution for each intervention. In addition, these parameters may aid in predicting the efficacy of non-pharmacological treatment.

Previous studies have indicated that characteristic impairments across cognitive domains (Mori et al., 2016), Barthel Index (Formiga et al., 2010), and lower pre-treatment regional cerebral blood flow in the right orbitofrontal cortex (Hongo et al., 2008) can be used to predict better responses to cholinesterase inhibitors treatment in older adults with dementia. Our findings suggest that the efficacy of non-pharmacological treatment can also be predicted based on neural characteristics and the extent of cognitive decline.

The present study has some limitations of note. First, as we did not include healthy controls, further studies are required to investigate differences in neuropsychological factors and regional brain atrophy between healthy individuals and participants with mild-to-moderate dementia. Such findings may allow clinicians to predict the most appropriate non-pharmacological treatment for each participant. Second, the intervention period of the present study lasted only 6 months. Explicit differences in neuropsychological factors and regional brain atrophy may be more evident following a longer intervention period. Future studies should also aim to include a larger number of participants to enhance the accuracy of prediction.

## Conclusion

Our findings suggest that participants with mild-to-moderate dementia who have experienced cognitive decline, reduced ability to perform activities of daily living, and extensive cortical atrophy are less likely to exhibit IMPs in cognitive function following non-pharmacological treatment. Thus, some characteristics of pre-treatment cognitive dysfunction and regional brain atrophy may aid clinicians in determining the most appropriate non-pharmacological intervention for each participant.

## Author Contributions

MS: conceived and designed the experiments. TT, NN, and KN: conducted the experiments. KT: analyzed the data. KT and MS: wrote the paper. JO: contributed materials. HK: analyzed and interpreted the data. HT: supervised and interpreted the data. All authors read and approved the final version of the paper.

## Conflict of Interest Statement

The authors declare that the research was conducted in the absence of any commercial or financial relationships that could be construed as a potential conflict of interest.

## Footnotes

1 http://imaging.mrc-cbu.cam.ac.uk/imaging/MniTalairach

## Abbreviations

AD: Alzheimer’s disease
CS: cognitive stimulation
ExM: exercise with music
GM: gray matter
IMP: improvement
LM: logical memory
MMSE: Mini-Mental State Examination
no-IMP: no-improvement
RBMT: Rivermead Behavioural Memory Test
RCPM: Raven’s Colored Progressive Matrices
VaD: vascular dementia
WF: word fluency.
